# A Novel ALDH1A1 Inhibitor Blocks Platinum-Induced Senescence and Stemness in Ovarian Cancer

**DOI:** 10.3390/cancers14143437

**Published:** 2022-07-15

**Authors:** Vaishnavi Muralikrishnan, Fang Fang, Tyler C. Given, Ram Podicheti, Mikhail Chtcherbinine, Tara X. Metcalfe, Shruthi Sriramkumar, Heather M. O’Hagan, Thomas D. Hurley, Kenneth P. Nephew

**Affiliations:** 1 Cell, Molecular and Cancer Biology Graduate Program, Medical Sciences Department, Indiana University School of Medicine, Bloomington, IN 47405, USA; vmuralik@iu.edu (V.M.); tygiven@iu.edu (T.C.G.); txmetcal@iu.edu (T.X.M.); ssriramkumar@wistar.org (S.S.); hmohagan@indiana.edu (H.M.O.); 2 Department of Medical and Molecular Genetics, Indiana University School of Medicine, Indianapolis, IN 46202, USA; ffang@indiana.edu; 3 Center for Genomics and Bioinformatics, Indiana University, Bloomington, IN 46202, USA; mnrusimh@indiana.edu; 4 Department of Biochemistry and Molecular Biology, Indiana University School of Medicine, Indianapolis, IN 46202, USA; mchtcher@iu.edu; 5 Indiana University Melvin and Bren Simon Comprehensive Cancer Center, Indianapolis, IN 46202, USA; 6 Department of Anatomy, Cell Biology and Physiology, Department of Obstetrics and Gynecology, Indiana University School of Medicine, Indianapolis, IN 46202, USA

**Keywords:** ovarian cancer, ALDH1A1, cancer stem cells, senescence, chemotherapy resistance

## Abstract

**Simple Summary:**

Ovarian cancer is the deadliest amongst the gynecologic malignancies. Most ovarian cancer patients initially respond to chemotherapy but will eventually relapse and become chemoresistant. A specialized subpopulation of cells within the tumor known as cancer stem cells are known to contribute to recurrence and chemoresistance. Ovarian cancer stem cells have a high expression of ALDH1A1, and patients with a high level of ALDH1A1 in their tumor have worse survival. Thus, specifically targeting ALDH1A1 could be an effective strategy to inhibit cancer stemness and disease relapse. We describe the discovery of a novel ALDH1A1 inhibitor called **974** and show that targeting ALDH1A1 with **974** decreases the population of ovarian cancer stem cells. Furthermore, inhibiting ALDH1A1 suppresses chemotherapy-induced senescence and stemness. Collectively, our data demonstrate that targeting ALDH1A1 in cancer stem cells could be an effective strategy to overcome chemotherapy resistance in ovarian cancer.

**Abstract:**

Ovarian cancer is a deadly disease attributed to late-stage detection as well as recurrence and the development of chemoresistance. Ovarian cancer stem cells (OCSCs) are hypothesized to be largely responsible for the emergence of chemoresistant tumors. Although chemotherapy may initially succeed at decreasing the size and number of tumors, it leaves behind residual malignant OCSCs. In this study, we demonstrate that aldehyde dehydrogenase 1A1 (ALDH1A1) is essential for the survival of OCSCs. We identified a first-in-class ALDH1A1 inhibitor, compound **974**, and used **974** as a tool to decipher the mechanism of stemness regulation by ALDH1A1. The treatment of OCSCs with **974** significantly inhibited ALDH activity, the expression of stemness genes, and spheroid and colony formation. An in vivo limiting dilution assay demonstrated that **974** significantly inhibited CSC frequency. A transcriptomic sequencing of cells treated with **974** revealed a significant downregulation of genes related to stemness and chemoresistance as well as senescence and the senescence-associated secretory phenotype (SASP). We confirmed that **974** inhibited the senescence and stemness induced by platinum-based chemotherapy in functional assays. Overall, these data establish that ALDH1A1 is essential for OCSC survival and that ALDH1A1 inhibition suppresses chemotherapy-induced senescence and stemness. Targeting ALDH1A1 using small-molecule inhibitors in combination with chemotherapy therefore presents a promising strategy to prevent ovarian cancer recurrence and has the potential for clinical translation.

## 1. Introduction

Ovarian cancer is the most fatal gynecological malignancy [1]. In the US, ovarian cancer was the fifth leading cause of death among women and worldwide it accounted for over 200,000 deaths in 2020 [2]. High-grade serous (HGS) is the most widely diagnosed subtype and accounts for 70–80% of ovarian cancer deaths [3]. Cytoreductive surgery and combination platinum-based chemotherapy have remained the mainstays of treatment. Although the majority of patients initially respond to chemotherapy, disease recurrence is common, and long-term survival in late-stage disease has improved little over the last four decades [4]. Mounting evidence shows that a small subpopulation of cells known as cancer stem cells (CSCs) are associated with tumor relapse and chemoresistance in ovarian [5,6] and other cancers [7]. Thus, it is essential to develop strategies to target CSCs in conjunction with conventional therapies.

CSCs are characterized by asymmetric division, i.e., the ability to self-renew as well as to differentiate into non-CSCs, resistance to chemotherapy and radiation, and the ability to survive without attachment. CSCs are identified by biomarkers such as CD133 [8], CD44/CD117 [9], and LGR5 [10] or the overexpression of aldehyde dehydrogenase (ALDH) enzymes [11]. ALDH1A1 is a member of the ALDH family and is highly expressed by stem cells in ovarian and other cancers [12]. Ovarian cancer cells with increased ALDH1A1 expression have a higher self-renewal ability [13], and HGSOC patients with tumors expressing high ALDH1A1 have poor overall survival [11]. Although ALDH1A1 is a well-accepted marker for OCSC, the exact mechanism by which ALDH1A1 regulates stemness remains incompletely understood.

Stemness can be promoted by cellular senescence [14,15]. Senescence is a cellular state of irreversible growth arrest induced by oncogenic activation or DNA-damaging therapies [16]. Senescent cells exhibit a complex secretome known as the senescence-associated secretory phenotype (SASP) that consists of cytokines, chemokines, and other growth factors. Senescence was initially thought to be tumor-suppressive; however, recent evidence suggests that senescent cells have a protumorigenic function [17]. In OC, platinum-based chemotherapy was shown to induce the CSC phenotype [18,19], and residual tumors after platinum treatment were enriched with ALDH+ cells [20]. Furthermore, platinum was shown to promote ovarian cancer stemness by paracrine signaling via SASP [21,22], which could contribute to CSC enrichment.

To study the functional role of ALDH1A1 in OCSC, we identified a specific small-molecule inhibitor, compound **974** (hereafter referred to as **974**). This inhibitor acts as a unique tool to selectively block ALDH1A1 activity over other ALDH isoforms. We demonstrated that **974** inhibited stemness phenotypes in ovarian cancer cell lines expressing ALDH1A1, and an in vivo limiting dilution analysis demonstrated an essential role for ALDH1A1 in CSC survival. Furthermore, the transcriptomic sequencing of **974**-treated HGSOC cells showed a downregulation of pathways related to stemness and chemoresistance, including NFκB, IL6 signaling, xenobiotic metabolism, drug efflux, and senescence, and **974** treatment blocked chemotherapy-induced senescence and stemness. These results suggest, for the first time, a novel role for ALDH1A1 in the maintenance of stemness via chemotherapy-induced senescence in OC.

## 2. Materials and Methods

### 2.1. Chemical Reagents

Compounds purchased from ChemDiv Corporation (San Diego, CA, USA) and ChemBridge Corporation (San Diego, CA, USA) were >95% pure based on vendor specifications (NMR spectra for the compounds can be found in the ). Compound **974** was resynthesized in the IU Chemical Genomics Core facility (IU School of Medicine, Indianapolis, IN, USA), was determined to be more than 99% pure by LC/MS, and its structure was validated by NMR.

### 2.2. Protein Purification and Enzymatic Assays

Human ALDH1A1, ALDH1A2, and ALDH1A3 were prepared and purified as previously described [23,24,25,26]. The inhibition of ALDH activity by compounds and the IC50 curves were determined by measuring the formation of NAD(P)H spectrophotometrically at 340 nm (molar extinction coefficient of 6200 M^−1^ cm^−1^) on a Beckman DU-640 as well as a Spectramax 340 PC spectrophotometer (GMI, Ramsey, NJ, USA) using a purified recombinant enzyme. The reaction components for assays with ALDH1A enzymes consisted of 100–200 nM enzyme, 200 µM NAD+, 100 µM propionaldehyde, and 1% DMSO in 25 mM BES buffer at pH 7.5. All assays were performed at 25 °C and were initiated by the addition of substrate after a 2 min incubation period. The purification of and the reaction conditions for the other ALDH isoenzymes were as described in [27]. The IC50 curves were collected for compounds that substantially inhibited ALDH1A activity at 20 µM. The data were fit to the four-parameter EC50 equation using SigmaPlot (v14), and the values represent the means/SEM of three independent experiments (n = 3).

### 2.3. X-ray Crystallography

All proteins used for crystallography were stored at −20 °C in 50% (*v/v*) glycerol. Before use, proteins were dialyzed exhaustively against 10 mM ACES, 1 mM DTT, and pH 6.6 buffer at 4 °C. Crystals were grown using the sitting drop geometry at 20 °C with crystallization solutions comprising pH 6.1–6.4 100 mM BisTris, 9–11% PEG3350 (Hampton Research, Catalog No. HR2-591, Aliso Viejo, CA, USA), 200 mM NaCl, and 5 mM YbCl3. The complex with CM38 was made by soaking apo-enzyme crystals for 5 h in the crystallization solution to which 500 µM compound in 2% DMSO (*v/v*) and 1 mM NAD+ had been added. The crystals were cryoprotected using 20% ethylene glycol (*v/v*) in the same ligand-soaking solution. Crystals were screened for diffraction on a Bruker X8 Prospector system (Bruker Corp., Billerica, MA, USA). Diffracting crystals were stored in liquid nitrogen for transport to the synchrotron source. Diffraction data were collected at Beamline 19-ID of the Advanced Photon Source (Argonne National Laboratory, Chicago, IL, USA). The data were integrated and scaled with the HKL3000 software suite. Rigid body, restrained TLS refinement, and structure validation were performed using PHENIX (v1.17, 2–4). Modeling and visualization were performed using Coot (v0.8.9.2, 5) within the PHENIX installation and PyMol v0.99 (DeLano Scientific LLC, San Francisco, CA, USA).

### 2.4. Cell Culture

The high-grade serous ovarian cancer ovarian cancer cell lines OVCAR3, OVCAR5, OVSAHO, and OVCAR8 were obtained from ATCC. Immortalized ovarian surface epithelial (IOSE) cells were a generous gift from Dr. Michael W. Y. Chan (National Chung Cheng University, Taiwan). The OVCAR5 cell line was maintained in DMEM (Gibco, Waltham, MA, USA, Catalog number: 11965092) with 10% FBS. All other cell lines were maintained in RPMI (Gibco, Waltham, MA, USA, Catalog no. 11875135) with 10% FBS, 10 mL of 100 mM sodium pyruvate (Thermo Fisher, Waltham, MA, USA, Catalog No. 11360070), and an antibiotic–antimycotic solution (Thermo Fisher, Waltham, MA, USA, Catalog No. 15240062). The cell lines were tested every 6 months for mycoplasma contamination using a Mycoalert kit (Lonza, Morristown, NJ, USA, Catalog No. LT07-318).

### 2.5. Flow Cytometry

The ALDH activity in live cells was measured by an ALDEFLUOR Assay (Stem Cell Technologies, Catalog No. 01700, Vancouver, BC, Canada) as per the manufacturer’s protocol. The percentage of ALDH+ cells was determined by an LSRII flow cytometer (BD Biosciences, Franklin Lakes, NJ, USA), using 488 nm excitation, and the signal was detected using the 530/30 filter. The ALDH+ percentage gate was determined by a sample-specific negative control diethylamino benzaldehyde (DEAB)/ALDH+ gate. CD133 was detected by flow cytometry using the fluorescent-labelled antibody CD133/2-PE (Miltenyi Biotec, Catalog No. 130-120-145, San Diego, CA, USA) in an LSRII flow cytometer using the filter 582/15. For each experiment, 10,000 events were analyzed. PI/Annexin V flow cytometry was performed using PI (ThermoFisher, Waltham, MA, USA, Catalog No. P1304MP) and Annexin V (ThermoFisher, Waltham, MA, USA, Catalog No. R37176). The flow cytometry data were collected using FACSDiva software (BD Biosciences, San Jose, CA, USA) and analyzed using FlowJo software (FlowJo LLC, Ashland, OR, USA).

### 2.6. Quantitative PCR

RNA was isolated from cultured cells using a RNeasy Mini Kit (Qiagen, Hilden, Germany, Catalog No. 74104) following the manufacturer’s protocol. Nanodrop (Thermo Fisher Scientific, Waltham, MA, USA) was used to determine RNA concentrations. qPCR was performed using a Lightcycler 480 Kit (Roche Diagnostics, Basel, Switzerland, Catalog No. 04707516001) using SYBR Green Roche 480 Light Cycler Master mix (Roche, Catalog No. 04887352001, Basel, Switzerland) as described previously [18]. All gene expression data were normalized to human EEF1A1. The relative expression levels were calculated using the 2^−ΔΔCt^ method. The primer sequences are provided in Supplementary Table S2.

### 2.7. Senescence Beta (β)-Gal Assay

Treated cells were stained for senescence-associated β-galactosidase activity according to the manufacturer’s protocol (Cell Signaling Technology, Danvers, MA, USA, Catalog No. 9860). The senescent cells were quantified by counting the stained cells from five independent fields, and the percentage was calculated based on the total number of cells in each field. Alternatively, the percentage of senescence-associated β-galactosidase cells was determined by flow cytometry using SPiDER-β-Gal (Dojindo, Rockville, MD, USA, Catalog No. SG-04) according to the manufacturer’s protocol.

### 2.8. RNA Sequencing and Bioinformatic Analysis

OVCAR3 cells were treated with compound **974** (5 µM) or DMSO for 48h in biological triplicates and total RNA was isolated using an RNeasy Mini Kit (Qiagen, Hilden, Germany, Catalog No. 74104) according to the manufacturer’s protocol. RNA-sequencing was performed essentially as we have described previously [28]. The RNA-seq results are available for download at the Gene Expression Omnibus (GEO) data repository at the National Center for Biotechnology Information (NCBI) under the accession number GSE200641. See the Supplementary Materials for a detailed description and bioinformatic analysis.

### 2.9. Mouse Xenograft Experiment

All mouse experiments were performed according to ethical guidelines approved by the Institutional Animal Care and Use Committee of Indiana University (Bloomington, IN, USA). For the limiting dilution analysis, 10^6^,10^5^, or 10^6^ OVCAR3 cells of indicated conditions were mixed with Matrigel (Corning, NY, USA, Catalog No. CLS356234) at a 1:1 ratio and injected subcutaneously into the right flanks of NOD SCID Gamma (NSG) mice. The tumor size was measured every week with a caliper, and the volume was calculated as ½*L*W2. At the end of the study, the tumors were collected and dissociated using a Tumor Dissociation Kit (Miltenyi Biotec, North Rhine-Westphalia, Germany, Catalog No.130-095-929) and a gentleMACS dissociator as per manufacturer’s protocol.

### 2.10. Colony Formation and Tumorsphere Assay

Cells at a 60–70% confluence in a 6 cm plate were treated for indicated times with the inhibitors. The cells were then collected by trypsinization and were plated as triplicates at a density of 500 cells/well in 24-well ultra-low-adherent plates (Corning, NY, USA, Catalog No. 3473) with 1 mL of stem cell medium as described previously in [18] for the spheroid formation assay or in 6-well plates in 2 mL of RPMI media with 10% FBS for the colony formation assay. Cells were allowed to grow for 7–14 days for spheroid formation or 5–7 days for colony formation. The spheroid size and morphology were assessed using a Zeiss Axiovert 40 inverted microscope with Axio-Vision software (Carl Zeiss Microimaging, Jena, Germany). Spheres larger than 10 mm were counted under the microscope. Colonies were stained with 0.5% crystal violet, and those with >50 cells were counted.

### 2.11. MTT Cell Proliferation Assay

Cells were collected after inhibitor treatments by trypsinization and then were seeded at a density of 2000 cells per well in 96-well plates, and a 3-(4.5-dimethylthiazol-2-yl)-2.5-diphenyl tetrazolium bromide (MTT; Thermo Fisher Scientific, Waltham, MA, USA, Catalog No. M6494) assay was performed during the day as described previously [29]. The optical density at 450 nm was measured using a BioTek Gen5 plate reader. The IC50 values were calculated using Prism 7 (GraphPad Software).

### 2.12. Cell Transfection and Plasmids

A total of 100,000 OVCAR3 cells were transfected with shControl (Sigma-Aldrich, St. Louis, MO, USA, MISSION shRNA lentiviralSHC001V) or shALDH1A1(Sigma-Aldrich, St. Louis, MO, USA, MISSION shRNA lentiviralTRCN0000026415 and TRCN0000026498) as described in [20]. Lipofectamine 2000 (Invitrogen, Waltham, MA, USA, Catalog No. 11668019) was used for transfection according to the manufacturer’s instruction. The lentivirus was produced as previously described to establish stable cell lines with ALDH1A1 knockdown [20].

### 2.13. Western Blotting

Cell lysates were prepared with RIPA lysis buffer (Thermo Fisher, Waltham, MA, USA, Cat No. 89900) or in 4% SDS in a QIAShredder (Qiagen, Hilden, Germany, Catalog No. 79656) containing Pierce^TM^ protease inhibitor cocktails and Pierce^TM^ phosphatase inhibitor. The protein concentration was measured by Bradford assay (Bio-Rad, Hercules, CA, USA, Catalog No. 5000001). Protein was loaded on precast 4–15% gels (Bio-Rad, Hercules, CA, USA), and standard blotting was performed. Primary antibodies for GAPDH, β-actin (Cell Signaling Technologies, Danvers, MA, USA), ALDH (BD Biosciences, San Jose, CA, USA, Catalog No. 611194.) were used. Membranes were incubated at 1:5000 with an HRP-conjugated secondary antibody (Cell Signaling Technologies, Danvers, MA, USA Catalog No. 7074). Proteins were visualized after incubation with the chemiluminescent substrate ECL (Pierce, Waltham, MA, USA, Catalog No. 32209).

### 2.14. Statistical Analysis

All data are presented as mean values ± SEM of at least three biological experiments unless otherwise indicated. Student’s *t* test was used to analyze the significant difference among different groups since the variation within the groups was similar. GraphPad Prism 7 software was used for data analysis and plotting.

### 2.15. Data Availability

The data generated in this study are available within the article and its supplementary materials files. The RNA-seq data generated in the study are publicly available in the Gene Expression Omnibus (GEO) under the accession number GSE200641.

## 3. Results

### 3.1. Discovery of ***974***, a Novel ALDH1A1-Specific Small-Molecule Inhibitor

Compound **974** (**974**) is an ALDH1A1-specific small-molecule inhibitor that was identified by screening compounds with high structural similarity to CM38, the lead compound identified from a high-throughput screen [27]. CM38 showed good structural characteristics as a lead compound, with a low molecular weight of 294 kDa and an approximate ClogP of 2.8. To investigate the nature of the interactions that define inhibition in this series of compounds, we determined the structure of ALDH1A1 in a complex with both NAD and CM38 by X-ray crystallography to a resolution of 1.8 Å (Supplementary Table S1, PDB ID: 7UM9). The structure of CM38 bound to ALDH1A1 showed that CM38 bound within the substrate binding pocket of the enzyme (Supplementary Figure S1A). CM38 was then screened for ALDH inhibition using nine ALDH isoenzymes at 20 µM and showed excellent selectivity for ALDH1A1 over the other highly similar isoenzymes in the ALDH subfamily (Supplementary Figure S1B). CM38 was found to be uncompetitive with respect to varied NAD+, which confirms that it does not bind the cofactor-binding site (Supplementary Figure S1C). There was no significant time-dependency in its ability to inhibit ALDH1A1, suggesting the interaction is non-covalent.

**Figure 1:**
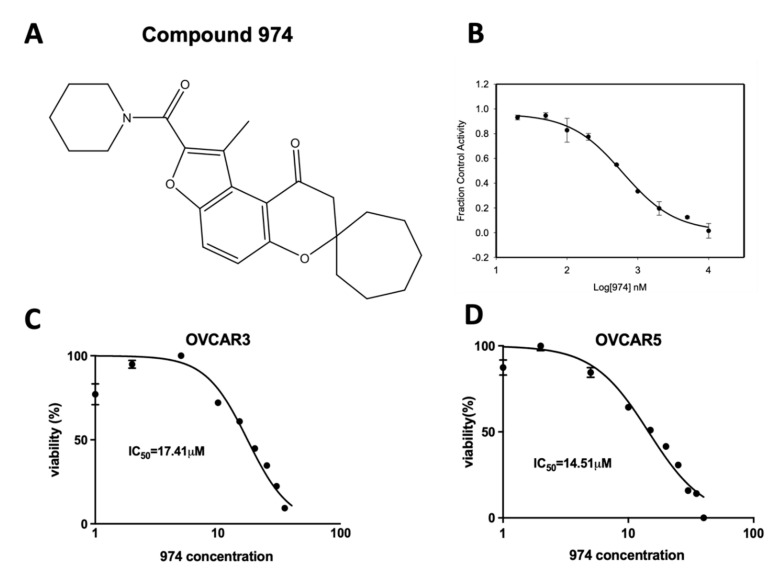
Compound **974**: A novel ALDH1A1 inhibitor. (**A**) Chemical structure of compound **974**. (**B**) EC50 curve for **974** binding with purified ALDH1A1. (**C**) OVCAR3 (**D**) OVCAR5 were treated with increasing doses of **974** (0.5–100 µM) for 48 h, and an MTT assay was performed to measure viability. IC_50_ values were calculated using GraphPad Prism.

To avoid the potential off-target effects due to the structure of CM38, **974** was chosen amongst the ALDH1A1 inhibitors with high structural similarity to CM38 from commercial sources (ChemDiv Corporation and ChemBridge Corporation, San Diego, CA, USA). The structure of **974** is shown in (Figure 1A). Further details about the discovery and characterization of **974** are described in the Supplementary Materials. It is a highly potent inhibitor that blocks ALDH1A1 activity with an IC50 of 470 nM (Figure 1B). The **974** doses chosen for the rest of the study were lower than the IC50 doses for OVCAR3 and OVCAR5 cells (Figure 1C).

### 3.2. ALDH1A1 Inhibition Suppresses Stemness Phenotypes in Ovarian Cancer Cells

**Figure 2:**
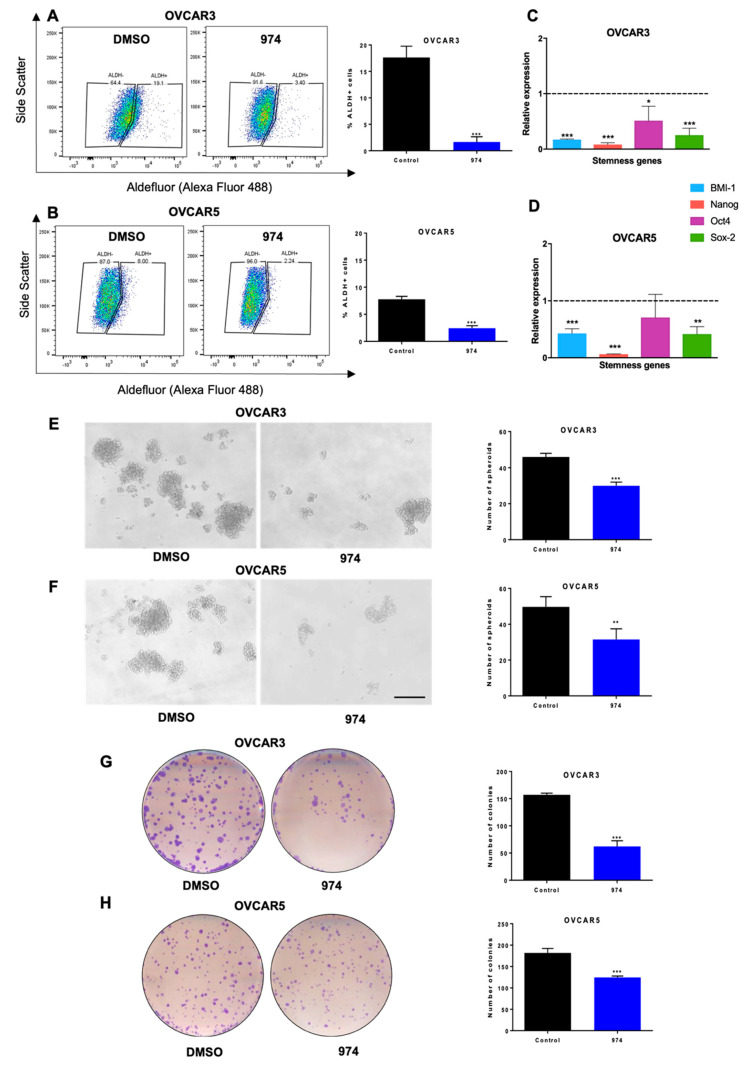
ALDH1A1 inhibition suppresses ovarian cancer stemness phenotypes in vitro. (**A**) OVCAR3 or (**B**) OVCAR5 cells were treated with compound **974** (5 µM for 48 h) or DMSO. The percentage of ALDH+ cells was measured by an ALDEFLUOR assay using flow cytometry (**left**), and the results were quantified (**right**). (**C**) OVCAR3 or (**D**) OVCAR5 cells were treated as in A, and the expression of stemness genes was measured by qPCR. (**E**) OVCAR3 or (**F**) OVCAR5 cells were treated as in (**A**), and 500 cells/well were replated in 24-well low-adhesion conditions after treatment. Representative images of spheroid formation after 14 days (**left**) and quantification (**right**). (**G**) OVCAR3 or (**H**) OVCAR5 cells were treated as in (**A**), and 500 cells/well were replated in 6-well plates after treatment. Colonies were stained with 0.05% crystal violet and counted. Representative images of colony formation (**left**) and quantification (**right**). Error bars represent SEM; n = 3 independent experiments of triplicate assays. Data are presented as means ± SEM with *p* < 0.05 (*), *p* < 0.01 (**), and *p* < 0.005 (***). Scale bar, 100 µm.

To test the effect of **974** on cellular ALDH enzyme activity, we performed ALDEFLUOR assays in HGSOC cell lines. It was found that **974** significantly reduced the percentage of ALDH-positive cells in OVCAR3 (Figure 2A) and OVCAR5 cells (Figure 2B). The gating strategy for the flow cytometry analysis for the ALDEFLUOR assay is shown in Supplementary Figure S2A. A dose–response study was performed to select the appropriate dose for the study. For this, the cells were treated with increasing doses of **974**, and ALDH activity was measured by an Aldefluor assay (Supplementary Figure S2B). At the tested doses, **974** did not affect the proliferation of ovarian cancer cells in a monolayer (Supplementary Figure S2C). At the doses of **974** selected for further study, the compound did not induce apoptosis, as indicated by PI/Annexin V staining (Supplementary Figure S2D). Moreover, **974** did not affect the proliferation of normal ovarian cells, indicating that it specifically targets cancer cells (Supplementary Figure S2E).

Numerous genes associated with stemness have been reported to be characteristic of OCSCs [30,31]. It was observed that **974** significantly decreased the expression of the well-known stemness genes Bmi-1, Nanog, Oct4, and Sox2 in OVCAR3 (Figure 2C) and OVCAR5 cells (Figure 2D). A spheroid assay was performed to measure the effect of **974** on the self-renewal ability of the CSC subpopulations at the time of plating. The spheroid formation ability of OVCAR3 (Figure 2E) and OVCAR5 cells (Figure 2F) was significantly inhibited by **974** treatment, and the clonogenic survival of both cell lines was also significantly inhibited by treatment with **974** (Figure 2G,H). To determine if the effects of **974** were specific to ALDH1A1-mediated stemness, we used OVCAR8 cells, which have relatively low ALDH activity [32] but have a CD133+ stem cell population (Supplementary Figure S3A). It was observed that **974** treatment did not alter clonogenic growth or spheroid formation in OVCAR8 cells (Supplementary Figure S3B–D). Treatment with **974** also did not alter the percentage of CD133 cells in OVCAR8 (Supplementary Figure S3A).

To determine if genetically reducing ALDH1A1 levels had similar effects on stemness properties as drug treatment, we developed two stable independent shRNA-mediated ALDH1A1 knockdown (shALDH1A1_1 and shALDH1A1_2) and scrambled control (shControl) OVCAR3 cell lines (Supplementary Figure S4A,B). ALDH1A1 knockdown significantly decreased the percent of ALDH+ cells, spheroid, and colony formation compared to shControl (Supplementary Figure S4C–E), similar to what was observed by **974** treatment. To test the specificity of **974** to ALDH1A1 in cells, shALDH1A1 and shControl cells were treated with **974**, and an ALDEFLUOR assay was performed. It was observed that **974** did not further reduce the percentage of ALDH+ cells in shALDH1A1 cells (Supplementary Figure S5).

### 3.3. ALDH1A1 Inhibition Suppresses Ovarian Cancer Stemness In Vivo

**Figure 3:**
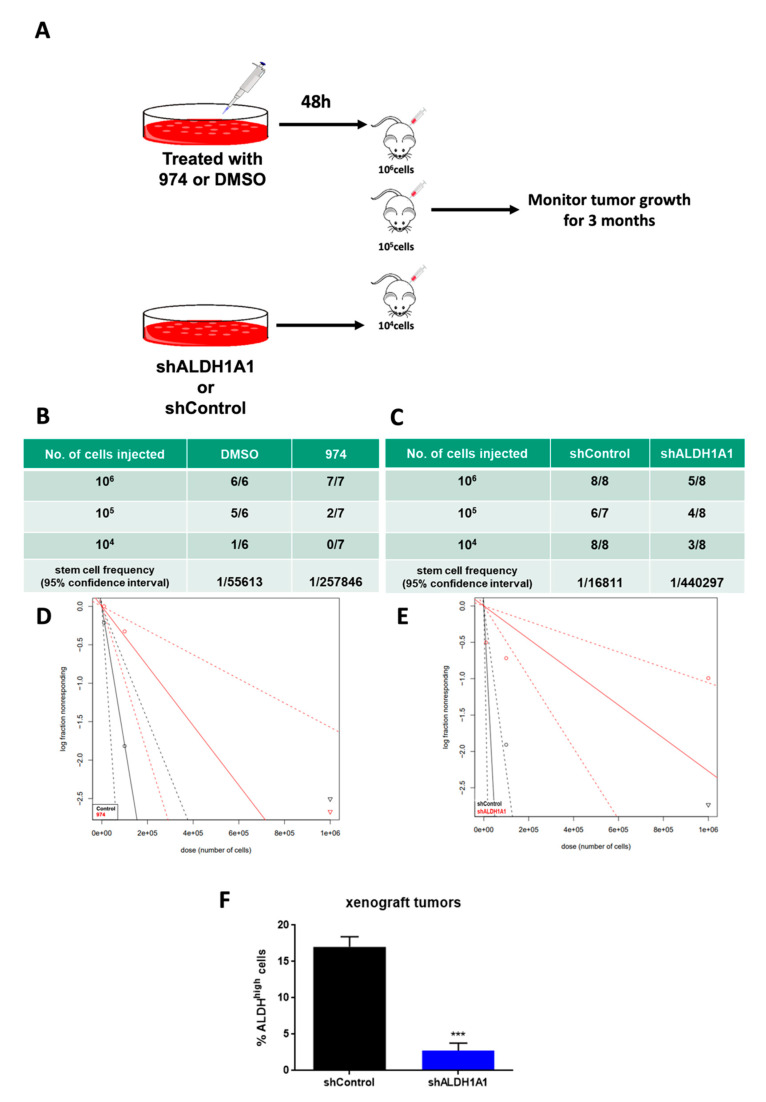
ALDH1A1 inhibition suppresses ovarian cancer stemness in vivo. (**A**) Schematic representing study design. Injections of 10^6^, 10^5^, or 10^4^ OVCAR3 cells treated with compound **974** (5 µM for 48 h), DMSO OR shALDH1A1, or shControl cells were given to NSG mice subcutaneously, and tumor formation was monitored. The numbers of mice with tumors over the total numbers of mice in the group and the CSC frequency were calculated by ELDA software (https://bioinf.wehi.edu.au/software/elda/, accessed on 12 May 2020) (**B**) for DMSO or **974** treatment and (**C**) for shControl or shALDH1A1. The log-fraction plot of limiting dilution analysis for stem cell frequency generated by extreme limiting dilution analysis in (**D**) compound **974** treatment vs DMSO or (**E**) shALDH1A1 or shControl. (**F**) The percentage of ALDH+ cells in the dissociated tumors from the shALDH1A1 study in (**C**) was measured by an ALDEFLUOR assay. Error bars represent SEM; n = 3 independent tumor samples. Data are presented as means ± SEM with *p* < 0.005 (***).

To test whether **974** treatment blocks tumor initiation in vivo, a limiting dilution analysis (LDA) was performed. OVCAR3 cells were pretreated with **974** (5 µM) or DMSO for 48 h; 1 million, 100,000, or 10,000 treated cells were injected subcutaneously (s.c.) into NSG mice; and tumor formation was monitored (Figure 3A). Treatment with **974** significantly reduced CSC frequency in mice (Figure 3B). Complementary to the study using **974** and to examine the requirement for ALDH1A1 in this context, shALDH1A1 or shControl cells (1 million, 100,000, or 10,000) were injected s.c in NSG mice. The results of the LDA demonstrated a significant reduction in CSC frequency (Figure 3C). The log fraction plot was generated using the LDA software for the **974** (or DMSO) study as well as the shALDH1A1 (or shControl) study (Figure 3D,E). The slope of the solid line represents the log-active cell (CSC) fraction. The 95% confidence interval is shown by the dotted lines. At the end of the study, tumors from the ALDH1A1 knockdown study were collected and analyzed for the percentage of ALDH+ cells by an ALDEFLUOR assay. The tumors from mice injected with shALDH1A1 cells had a significantly lower percentage of ALDH+ cells compared to the tumors from shControl-injected mice (Figure 3F). Collectively, these data demonstrate that ALDH1A1 is essential for the maintenance of stemness in ovarian cancer cells and that **974** significantly inhibits stemness phenotypes.

### 3.4. ALDH1A1 Inhibition Downregulates Key Stemness and Chemoresistance Pathways

**Figure 4:**
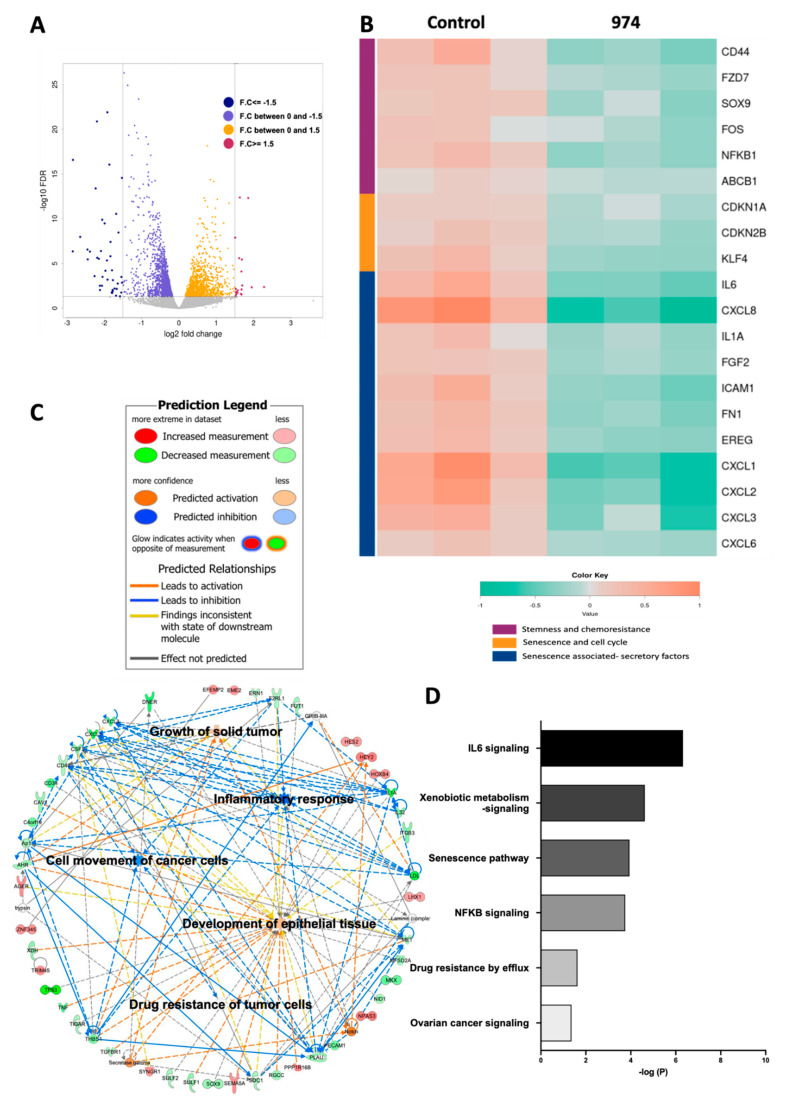
ALDH1A1 inhibition suppresses pathways involved in chemoresistance and stemness. RNA-seq was performed on OVCAR3 cells treated with compound **974** (5 µM for 48 h) or DMSO (n = 3). (**A**) Volcano plot of genes up and downregulated by ALDH1A1 inhibition. (**B**) Heatmap of selected genes significantly downregulated by compound **974** (FDR < 0.05). (**C**) Networks of biological processes constructed using significantly altered genes (FDR < 0.05) between OVCAR3 cells treated with **974** or DMSO. (**D**) Canonical pathways related to stemness and chemoresistance identified by the Ingenuity Pathway Analysis (IPA) using genes significantly altered by **974** treatment (FDR < 0.05, fold change > |1.5|).

To determine the effect of ALDH1A1 inhibition by **974** on gene expression, a transcriptomic analysis of **974** or DMSO treated cells was carried out using RNA-seq and a bioinformatic analysis. The volcano plot shows that 1630 genes were downregulated, and 1140 genes were upregulated by ALDH1A1 inhibition (Figure 4A, **974**- vs. DMSO-treated samples; FDR < 0.05). Genes significantly downregulated by ALDH1A1 inhibition included stem cell markers (CD44, FZD7, and SOX9) and genes involved in chemoresistance (ABCB1 and NFκB) in ovarian cancer [9,33,34,35] (Figure 4B). In addition, **974** treatment significantly downregulated the senescence biomarkers p21(CDKN1A) and p15INK4b (CDKN2B) and genes associated with the senescence-associated secretory phenotype (SASP), including IL6, IL8, CXCL1, and CXCL3 (Figure 4B). The Ingenuity Pathway Analysis (IPA) and Gene Ontology (GO) annotations of the differentially expressed genes demonstrated that **974** treatment inhibited a number of key biological processes associated with tumor initiation and stem cells, including the growth of solid tumor, the inflammatory response, the movement of cancer cells, the development of epithelial tissues, and the drug resistance of tumor cells (red and green colors represent upregulated or downregulated genes, respectively), supporting the role of ALDH1A1 in modulating OCSC biology (Figure 4C). The IPA of genes significantly decreased by **974** treatment revealed altered xenobiotic metabolism signaling, cancer drug efflux, and IL6 and NFκB signaling (Figure 4D, Supplementary Table S4), all of which have been reported to play a role in stemness and chemoresistance [20,36,37]; furthermore, the cellular senescence pathway was significantly downregulated by **974** treatment (Figure 4D). Collectively, these results demonstrated that ALDH1A1 inhibition led to a reduction in gene expression in stemness- and chemoresistance-related pathways in OC.

### 3.5. Inhibition of ALDH1A1 Suppresses Chemotherapy-Induced Senescence and Stemness

**Figure 5:**
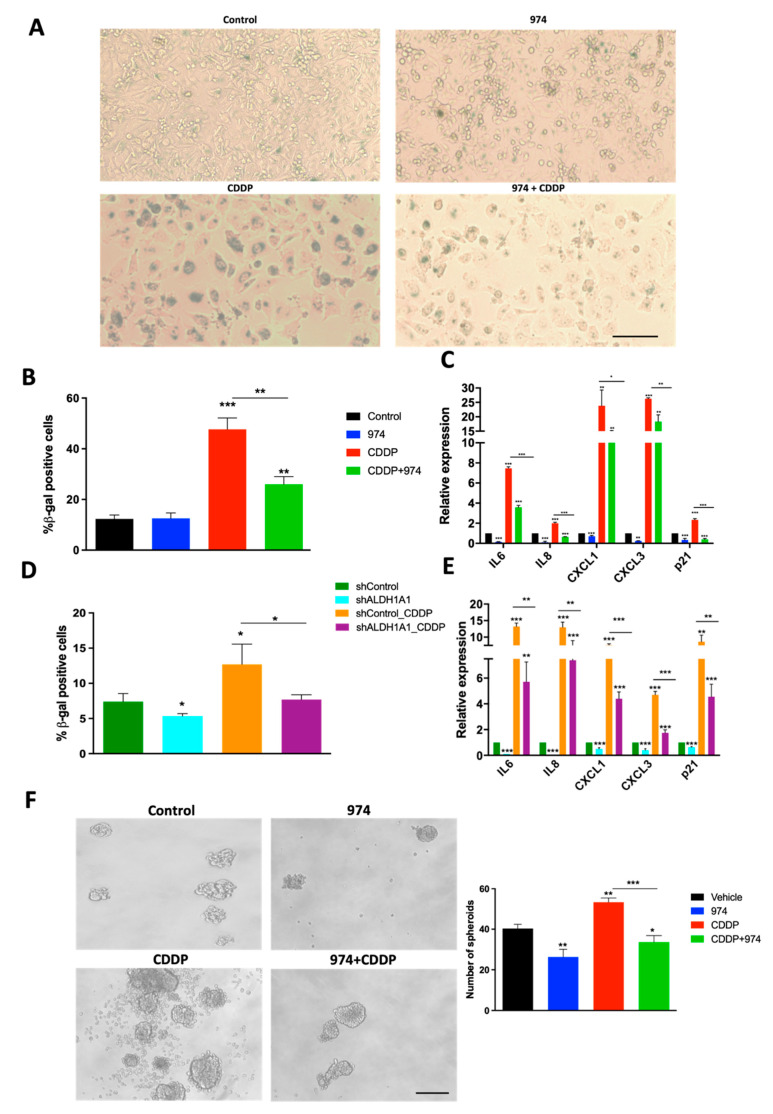
ALDH1A1 inhibition suppresses chemotherapy-induced senescence and stemness in HGSOC cells. (**A**) Senescence-associated (SA) beta-gal assay was performed on OVCAR3 cells treated with DMSO, compound **974** (5 µM for 48 h), cisplatin (CDDP) (15 µM for 16 h), or both. Representative images at 10× magnification. (**B**) Quantification of SA-beta-gal assay represents percentage of senescent cells averaged from five different fields in each condition. (**C**) Expression of SASP genes and p21 (CIP1/WAF1) was examined by qPCR in OVCAR3 cells treated as in (**A**). (**D**) Percentage of SA-beta-gal-positive cells in shControl or shALDH1A1 cells treated with NaCl (vehicle) or CDDP (15 µM for 16 h) was measured by flow cytometry using Spider beta gal reagent. (**E**) Expression of p21 and SASP genes was examined by qPCR in shControl or shALDH1A1 cells treated with NaCl or CDDP (15 µM for 16h). (**F**) OVCAR3 cells treated with DMSO, compound **974** (5 µM for 48 h), cisplatin (CDDP) (7.5 µM; _½_ IC50 for 3 h), or both were plated in low-attachment conditions at a density of 500 cells/well. Representative spheroid images captured on Day 7 (**left**). Quantification of spheroids (**right**). Error bars represent SEM; n = 3 independent experiments of triplicate assays. Data are presented as means ± SEM with *p* < 0.05 (*), *p* < 0.01 (**), and *p* < 0.005 (***). Scale bar, 100 µm.

Senescence is the cellular state characterized by proliferative arrest, resistance to apoptosis, and an altered expression of genes encoding cytokines and other growth factors, commonly known as SASP [38]. SASP has protumorigenic paracrine effects, and emerging evidence supports the role of SASP in the induction of cancer stemness and relapse [17,22]. Because platinum-based chemotherapy has been shown to enhance SASP and subsequently stemness in ovarian cancer [22], we investigated the effect of ALDH1A1 inhibition on senescence in cisplatin (CDDP)-treated cells. Treatment with **974** suppressed CDDP-induced senescence-associated beta galactosidase (SA-β gal) staining in OVCAR3 cells (Figure 5A,B). The **974** treatment also significantly reduced the basal and CDDP-induced expression of senescence marker p21 (CDKN1A) in OVCAR3 cells (Figure 5C) and SASP gene expression in OVCAR3 and OVCAR5 cells (Figure 5C; Supplementary Figure S6A). Additionally, **974** significantly inhibited SASP gene expression in CDDP-resistant OVCAR5 cells with high ALDH activity, which was developed by repeated cycles of exposure to CDDP (Supplementary Figure S6B–D, Supplementary Materials). The effect of ALDH1A1 inhibition on the senescence phenotype was validated using shALDH1A1 cells. ALDH1A1 knockdown significantly suppressed the basal and CDDP-induced senescence, which were measured as the percentage of β-gal-positive cells (Figure 5D). ALDH1A1 knockdown suppressed the basal and CDDP-induced expression of p21 and SASP genes, including IL6, IL8, CXCL1, and CXCL3 (Figure 5E).

CDDP induces stemness in ovarian cancer cells and treatment with **974** abrogated the CDDP-induced stemness phenotype in spheroid assays (Figure 5F), suggesting a link between senescence and stemness. To confirm that blocking senescence reduced stemness, ovarian cancer cells were treated with ABT-263, a senolytic agent (Navitoclax; 2 µM for 24 h). ABT-263 treatment reduced basal and CDDP-induced spheroid numbers (Supplementary Figure S6C).

## 4. Discussion

Ovarian cancer is a deadly disease attributed to late-stage detection as well as relapse and the development of chemoresistance. Strategies to overcome chemoresistance are needed to achieve a better prognosis in ovarian cancer patients. OCSCs have been shown to cause chemoresistance [18]. Thus, targeting this population in conjunction with conventional chemotherapy could be an effective strategy for preventing relapse. This study describes the discovery and characterization of compound **974**, a novel small-molecule inhibitor selective to ALDH1A1 over other ALDH isoforms. We show that **974** inhibits stemness phenotypes in HGSOC cell lines, blocks the expression of putative stemness genes and pathways, reduces OCSC frequency, and delays tumor initiation in vivo. Importantly, the inhibition of ALDH1A1 by **974** suppresses platinum-based chemotherapy-induced senescence and stemness, and to our knowledge, this is the first report that ALDH1A1 regulates senescence-mediated stemness. Overall, our findings support the use of small-molecule inhibitors of ALDH1A1 as a promising therapeutic approach to target OCSC and prevent chemoresistance.

ALDH1A1 is a robust marker for CSCs in ovarian and other cancers [12,39], and ALDH1A1 expression in patient tumors predicts poor prognosis [11,40,41]. Landen et al. demonstrated that in an orthotopic ovarian cancer mouse model, ALDH1A1 silencing by siRNA sensitized both paclitaxel- and carboplatin-resistant cell lines to chemotherapy and inhibited tumor growth significantly. Additionally, ALDH1A1 has been shown to contribute to PARP inhibitor resistance in ovarian cancer, and ALDH1A1 inhibition synergizes with the PARP inhibitor olaparib in killing BRAC2-mutated ovarian cancer cells [14]. These data provide support for targeting ALDH1A1 in ovarian cancer patients to overcome chemotherapy resistance [11]. Several inhibitors have been designed to target CSCs by the selective inhibition of ALDH1A1 or by a pan-ALDH1A inhibition approach. Pan-ALDH1A inhibitors have the advantage of targeting multiple ALDH isoforms at once. The pan-ALDH1A inhibitor 673A causes cell death by necroptosis in OCSCs, reduces tumor initiation, and is highly synergistic with chemotherapy. Disulfiram, another broad ALDH inhibitor, was shown to have better efficacy in inhibiting CSC populations than the ALDH1A1-specific inhibitors NCT-505 and NCT-506 in cells including the OVCAR8 cell line with low ALDH1A1 levels [32]. However, targeting ALDH1A1 selectively could have a unique advantage from a safety perspective because ALDH1A1 has been shown to be dispensable for stem cell function in mice [42]. Moreover, ALDH1A2 expression is essential for dendritic cell differentiation in the bone marrow microenvironment [43]. Even though ALDH1A3 expression is elevated in primary ovarian cancer [44], high ALDH1A1 in tumors is correlated with a poor prognosis in patients, suggesting that ALDH1A1 is the more important target for improved survival [11]. Our current study demonstrates that ALDH1A1 inhibition suppresses stemness and alleviates chemoresistance. Future work will investigate whether ALDH1A1 inhibition leads to compensation by other ALDH isoforms.

The lead compound, CM38, on which **974** is based, targets a novel scaffold in ALDH1A1 compared to the previously published ALDH1A1 inhibitors CM37 [13] or CM39 [45]. By targeting a novel scaffold in the ALDH1A1 substrate binding pocket, **974** acts an effective tool for further understanding ALDH1A1 function. We show that **974** is specific to ALDH1A1 by demonstrating no change in stemness when treating a low ALDH1A1 expression cell line with **974** (OVCAR8 cells; Supplementary Figure S3A–D). Moreover, when ALDH1A1 is biologically inhibited using a knockdown, **974** can no longer inhibit ALDH activity (Supplementary Figure S5). The effects are in line with the published reports of other ALDH1A1-specific inhibitors, CM37 [13] and NCT-501 [46]. The data on ALDH1A1 inhibition using CM37 and NCT-501 support our findings that ALDH1A1 is essential to maintain CSC phenotypes. CM37 inhibited spheroid formation and the expression of stemness genes such as Sox2, Nanog, and Oct4 as well as p21 similar to **974** and the biological knockdown of ALDH1A1 [13,47]. NCT-501 inhibits ALDH activity and attenuates the de-differentiation of non-CSCs into CSCs in ovarian cancer cells [46]. ALDH1A1 has also been indirectly targeted by the inhibition of upstream ALDH1A1 regulators such as BRD4 by the (bromodomain and extra terminal) BET inhibitor JQ1 [48] or HOTAIR by PNA3 [49], resulting in reduced ALDH1A1 expression, which leads to the inhibition of stemness. Although **974** is not yet formulated as an in vivo therapeutic, we provide compelling evidence using ovarian cancer cells treated with **974** in vitro for LDA as support for future rational chemistry design strategies to improve **974** bioavailability and targeting CSC in vivo. Our ongoing efforts aim at targeting the “arms” that extend from the central scaffold to improve the metabolic stability and modify the lipophilicity of the compound.

ALDH1A1 is a ubiquitous enzyme with several cellular functions such as the conversion of aldehydes into carboxylic acids, scavenging ROS, and altering signaling through the modulation of the retinoic acid pathway [12]. Thus, how ALDH1A1 regulates cancer stemness could involve more than one mechanism. Through transcriptomic analysis, our study reveals a previously unknown mechanism of stemness regulation by ALDH1A1 via the senescence pathway. Senescence, initially thought to be a tumor-suppressive mechanism, has recently been shown to promote stemness in ovarian [22] and other cancers [17]. Senescent cells exhibit a complex secretome known as SASP that promotes stemness via paracrine signaling [22,50]. Specifically, the IL-6 signaling axis was shown to be upregulated by neo-adjuvant chemotherapy [51]. We demonstrate that ALDH1A1 inhibition blocks the senescence and SASP induced by cisplatin treatment (Figure 5), possibly by suppressing the NFκB pathway (Figure 4D). Consistent with our findings, the broad ALDH inhibitor disulfiram was shown to inhibit cancer stemness via the NFκB pathway [32]. NFκB regulates several of the SASP factors [39], and inhibiting NFκB attenuates stemness in ovarian cancer in vitro as well as in vivo [19,52]. Further investigation is required to elucidate the exact mechanism by which ALDH1A1 regulates cisplatin-induced stemness.

In conclusion, using **974** as a tool, we have demonstrated the functional significance of ALDHA1 in maintaining ovarian cancer stemness in in vitro and in vivo models. To our knowledge, this is the first study that demonstrates that ALDH1A1 is involved in the regulation of senescence and SASP. ALDH1A1 regulation of senescence could be significant because the standard-of-care treatment for ovarian cancer includes platinum-based chemotherapy, and carboplatin has been shown to induce senescent cells in ovarian cancer tumors [53,54]. We show that a new isoform-specific ALDH1A1 inhibitor suppresses chemotherapy-induced senescence as well as stemness. Targeting ALDH1A1 in combination with chemotherapy could block senescence and inhibit CSC enrichment to overcome resistance and improve outcomes for ovarian cancer patients.

## 5. Conclusions

In this study, we describe the discovery of compound **974**, a novel small-molecule inhibitor specific to ALDH1A1. Using compound **974** to target ovarian cancer stem cells, we show that ALDH1A1 plays a key role in cancer stemness. Additionally, compound **974** suppressed chemotherapy-induced senescence and stemness in ovarian cancer. Collectively, our data demonstrate that the inhibition of ALDH1A1 with a small-molecule inhibitor in combination with chemotherapy could suppress senescence and stemness to improve outcomes for ovarian cancer patients.

## Data Availability

The data generated in this study are available within the article and its supplementary materials files. The RNA-seq data generated in the study are publicly available in the Gene Expression Omnibus (GEO) under the accession number GSE200641.

## Supplementary Materials

The following supporting information can be downloaded at: https://www.mdpi.com/article/10.3390/cancers14143437/s1, Supplementary Figure S1: X-ray crystallography of CM38 with ALDH1A1 and characterization of inhibitor activity, Supplementary Figure S2: Gating strategy for Aldefluor assay, dose response for **974**, and proliferation and apoptosis assay data, Supplementary Figure S3: CD133 expression and stemness assay validation in OVCAR8, Supplementary Figure S4: ALDH1A1 expression and stemness assays in ALDH1A1 shRNA knockdown and scrambled control cell lines, Supplementary Figure S5: Aldefluor assay validation for ALDH1A1 knockdown and scrambled control cells treated with **974**, Supplementary Figure S6: SASP gene expression in OVCAR5, characterization of CDDP-resistant OVCAR5 cells and SASP expression and spheroid formation in CDDP and ABT-263 combination, Supplementary Table S1: CM38 X-ray crystallography data, Supplementary Table S2: Primer sequences used for qPCR, Supplementary Table S3: Compound activity against ALDH isoforms [55,56,57,58].
